# Network-based modeling of drug effects on disease module in systemic sclerosis

**DOI:** 10.1038/s41598-020-70280-y

**Published:** 2020-08-07

**Authors:** Ki-Jo Kim, Su-Jin Moon, Kyung-Su Park, Ilias Tagkopoulos

**Affiliations:** 1 Division of Rheumatology, Department of Internal Medicine, St. Vincent’s Hospital, College of Medicine, The Catholic University of Korea, Seoul, Republic of Korea; 2 Division of Rheumatology, Department of Internal Medicine, Uijeongbu St. Mary’s Hospital, College of Medicine, The Catholic University of Korea, Seoul, Republic of Korea; 3 St. Vincent’s Hospital, 93 Jungbu-daero, Paldal-gu, Suwon, Gyeonggi-do 16247 Republic of Korea; 4 Department of Computer Science, University of California, Davis, CA USA; 5 Genome Center, University of California, Davis, CA USA; 6 AI Institute for Next-Generation Food Systems, AIFS, Davis, CA USA

**Keywords:** Computational biology and bioinformatics, Systems biology, Rheumatology

## Abstract

The network-based proximity between drug targets and disease genes can provide novel insights regarding the repercussions, interplay, and repositioning of drugs in the context of disease. Current understanding and treatment for reversing of the fibrotic process is limited in systemic sclerosis (SSc). We have developed a network-based analysis for drug effects that takes into account the human interactome network, proximity measures between drug targets and disease-associated genes, genome-wide gene expression and disease modules that emerge through pertinent analysis. Currently used and potential drugs showed a wide variation in proximity to SSc-associated genes and distinctive proximity to the SSc-relevant pathways, depending on their class and targets. Tyrosine kinase inhibitors (TyKIs) approach disease gene through multiple pathways, including both inflammatory and fibrosing processes. The SSc disease module includes the emerging molecular targets and is in better accord with the current knowledge of the pathophysiology of the disease. In the disease-module network, the greatest perturbing activity was shown by nintedanib, followed by imatinib, dasatinib, and acetylcysteine. Suppression of the SSc-relevant pathways and alleviation of the skin fibrosis was remarkable in the inflammatory subsets of the SSc patients receiving TyKI therapy. Our results show that network-based drug-disease proximity offers a novel perspective into a drug’s therapeutic effect in the SSc disease module. This could be applied to drug combinations or drug repositioning, and be helpful guiding clinical trial design and subgroup analysis.

## Introduction

Cells must act like a well-orchestrated group, thus, genes, proteins and other chemical compounds seldom operate separately but instead act together to perform the various biological functions of the cellular network^1^. In the context of disease, various combinations of dysregulated genes converge to activate common pathways and perturb the shared network states, resulting in complex diseases with similar phenotypes^2,3^. Similarly, drugs exert their therapeutic effects by modulating molecular pathways or networks linked with their primary target molecule(s)^4^. It is also thought that system-wide molecular actions of drugs are partially responsible for off-target effects, adverse effects, or beneficial effects in off-label indications^5^. Direct study of the respective pathways and biological processes is cumbersome. However, systemic analysis of large integrated datasets has recently provided opportunities to reveal missing connections, and hence to bridge gaps in the existing knowledge, to better understand human diseases^6,7^. Using knowledge of the proximity of compounds and their targets to key disease genes has emerged as a possible method to identify potential compound-disease pairs. These analyses are based on the hypothesis that the closer a drug’s target is to the disease-associated network, the higher the chance it will influence the disease’s state and progression^8^. This principle can guide drug topology in the network module of the disease and the drug’s utilization in personalized medicine.

Systemic sclerosis (SSc) is a chronic autoimmune disease characterized by a distinctive pathogenetic triad of microvascular damage, dysregulated autoimmunity and generalized fibrosis of the skin and multiple internal organs, usually resulting in permanent functional impairment associated with high morbidity and mortality^9^. Current therapeutic approaches consist of treatments against the vasculopathy, general immunosuppression and organ-specific therapies for the affected organs, although no single therapy has yet proven to effectively reverse or alleviate the fibrotic process in SSc^9^. Many past clinical trials have produced negative results, which may be due to: being driven by the wrong choice of target pathways or molecules, the chronic and irreversible nature of the fibrosis, the lack of indices properly evaluating the treatment response, and the heterogeneity of the trial participants^10,11^. Hence there is a need for a systematic, in-depth view of the status of currently used or investigational drugs in the mechanistic network of disease. Analyzing drug action from the viewpoint of network biology may provide insights into how we can improve drug discovery and use in complex diseases such as SSc.

In this study, we analyzed the relationship between currently used and potential drugs and SSc-associated subnetworks, using proximity measures in an unsupervised and unbiased network-based framework. First, we explored the distance from key molecules, which are targeted by the drugs, to the SSc-associated genes. Then we concentrated on the pertinent pathways and associated biological processes, based on the distance metrics with a focus on the degree to which the drug-perturbed pathways cover pathogenic processes of SSc. Finally, we investigated how the drugs work to disturb the network of the disease module, validated the simulated drug actions in the gene-expression profiles of SSc skin tissue, and considered how this result could be applied to clinical research or practice.

## Results

### SSc-associated genes and enriched biological processes

**Figure 1:**
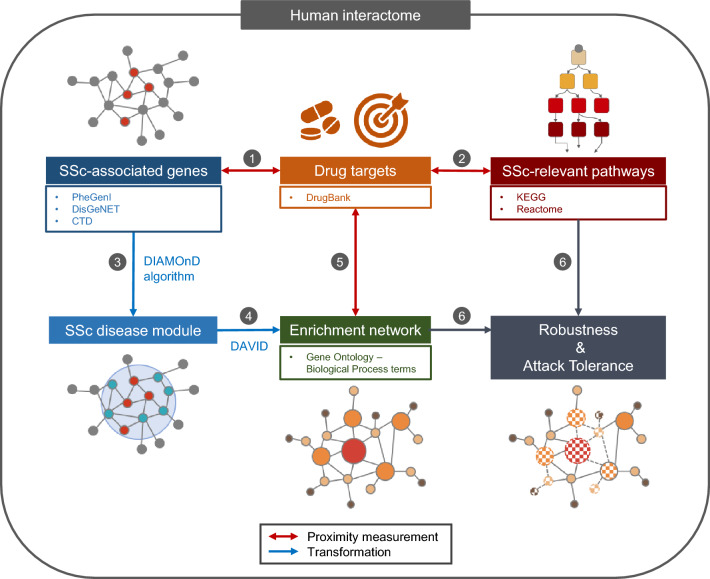
Overview of the computational approach, with network proximity data sources and methodology. Drug targets, SSc-associated genes and relevant pathways were curated from the literature. The SSc-associated genes were used as seeds for identification of the SSc disease module, using the DIAMOnD module-finding algorithm on the human interactome network. Once the SSc disease module had been constructed, GO analysis was performed with DAVID software, for assessment of the related biological processes. Network-based closest proximity between two molecular groups was calculated, and attack vulnerability of the disease module network by drugs was assessed.

**Figure 2:**
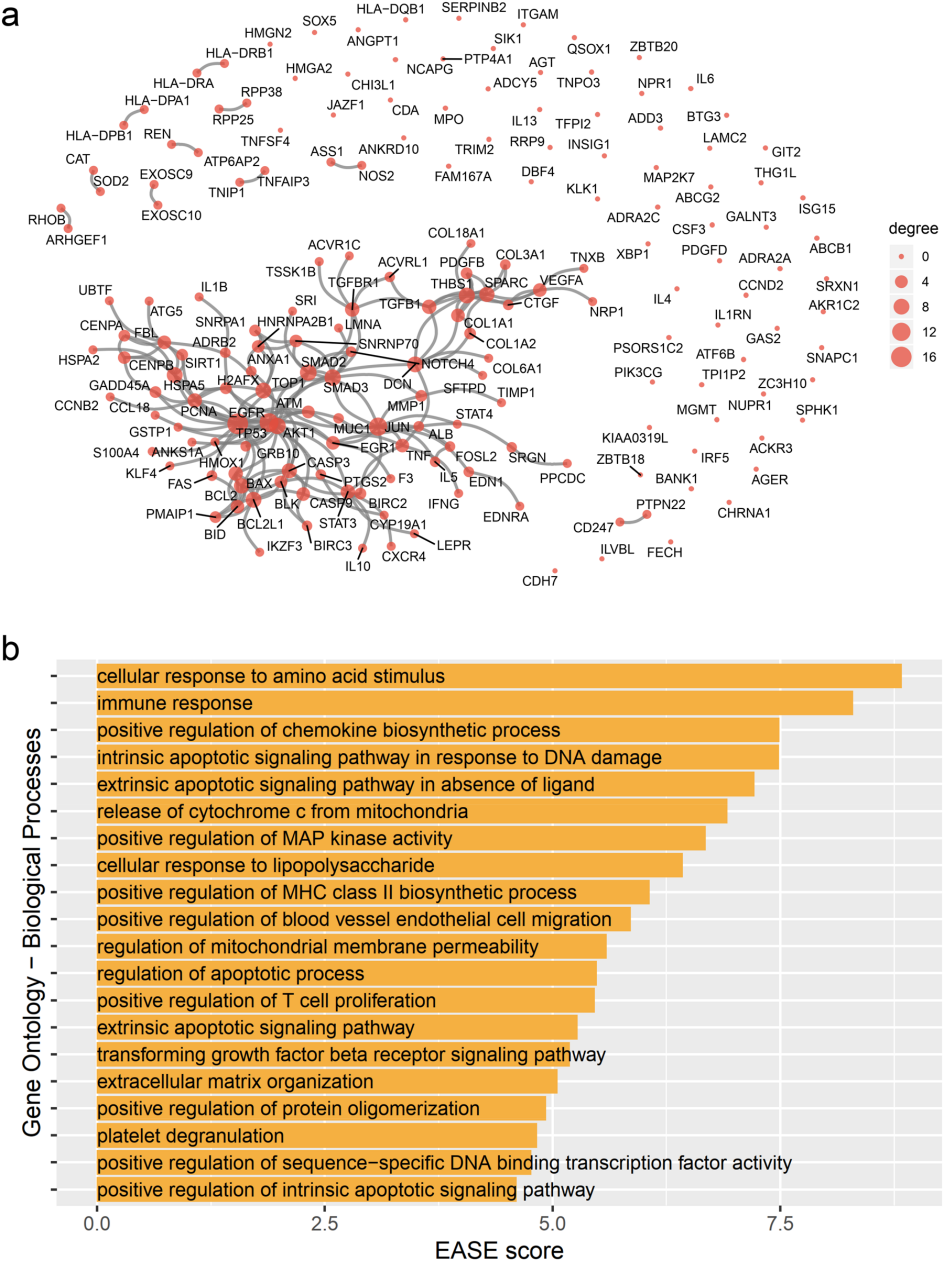
Proto-module with SSc-associated genes and their enriched processes. (**a**) A graphical representation of all the interactions between SSc-associated genes. The size of each node was determined by its degree. (**b**) Functional enrichment analysis was done using DAVID software, resulting in 55 gene ontology—biological process terms. The top 20 terms, by expression analysis systematic explorer (EASE) score, are shown.

The overarching methodology for our data collection and proximity analysis performed was depicted in Fig. 1. We identified the 179 SSc-associated genes from the Phenotype-Genotype Integrator (PheGenI)^12^, the DisGeNET^13^, and the Comparative Toxicogenomics Database (CTD)^14^ (Supplementary Table 1). In the human interactome network, the largest connected component (LCC) consisted of 88 SSc-associated genes, 20 genes that were paired off and 71 genes that were scattered (Fig. 2a). Enriched biological processes for the 179 SSc-associated genes were searched using the DAVID[The Database for Annotation, Visualization and Integrated Discovery] tool^15^, resulting in 55 gene ontology (GO)-biological process terms. Chemokine synthesis, apoptosis-associated processes, the transforming growth factor (TGF)-β signaling pathway, extracellular matrix organization, and some immune response-related processes (MHC class II biosynthesis process, T-cell proliferation) were captured as the main features (Fig. 2b). This result indicates that the list of genes and molecules studied to-date as involved in the pathogenesis of SSc is incomplete and that there are missing nodes and links.

### Proximity between the SSc-targeted drugs and SSc-associated genes in the interactome

**Figure 3:**
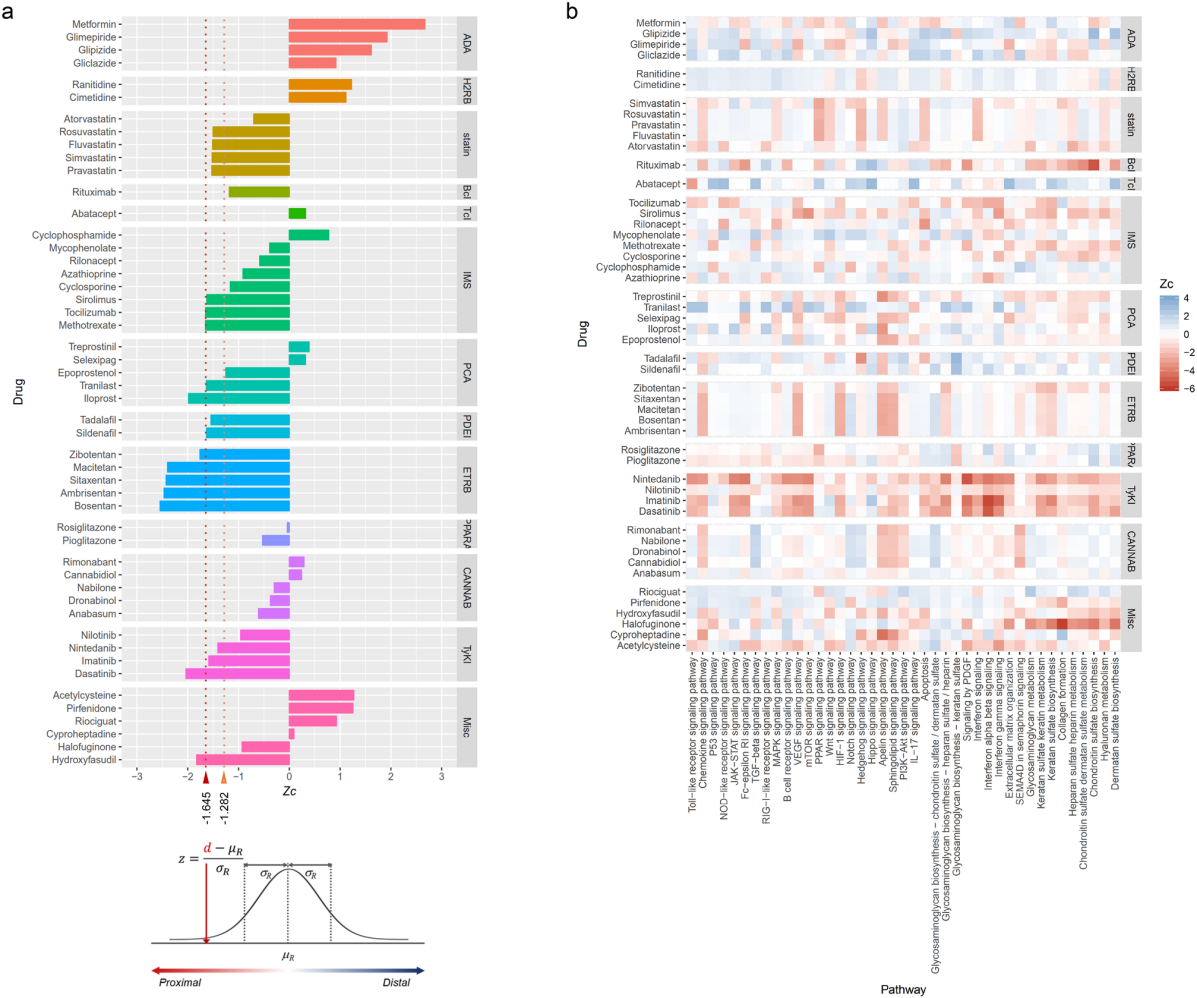
Proximity between drug targets and SSc-associated genes and pathways. (**a**) Given a set of disease proteins *S*, and the set of drug targets *T*, the closest distance *d* is the shortest path-length between all members of *S* and *T* in the network. The relative proximity (*z*) was calculated by comparing the distance *d*, between *T* and *S,* to a reference distribution of distances between SSc-associated proteins and the 10^3^ groups of randomly selected proteins matching the sizes and degrees of the drug targets in the network. The red dotted lines correspond to the significance thresholds. If *z*_*c*_ ≤  −1.645 (one-sided *P *value < 0.05), a drug was considered to be significantly proximal to the disease and if *z*_*c*_ ≤  −1.282 (one-sided *P *value < 0.10), a drug was considered to be probably proximal to the disease. (**b**) Heatmap of drugs and SSc-relevant pathways based on proximity metrics. ADA, anti-diabetic agent; H2RB, H2-receptor blockers; BcI, B-cell inhibitor; TcI, T-cell inhibitor; IMS, immunosuppressive agents; PCA, prostacyclin analogs; PDEI, phosphodiesterase-5 inhibitors; ETRB, endothelin receptor blockers; PPARA, PPAR-g agonists; CANNAB, cannabinoids; TyKI, tyrosine kinase inhibitors; Misc, miscellaneous drugs.

Currently used and potential drugs for SSc were identified from the literature^10,16–18^ and drug-target information was gathered from the DrugBank database^19^ (Supplementary Table 2). Anti-diabetic agents, H2 receptor blockers, and statins were compared as controls because these drugs are far from the mechanistic features of SSc. The relative proximity between the drugs and the SSc-associated genes is plotted in Fig. 3a. The relative proximity between the drugs and the SSc-associated genes is plotted in Fig. 3a. Anti-diabetic agents and H2-receptor blockers were located far from the SSc-associated genes, whereas statins were probably proximal to the SSc-associated genes (*z*_*c*_ <  −1.282, *P *value < 0.10). Rituximab, abatacept, peroxisome proliferator-activated receptor (PPAR)-γ agonists, and cannabinoids were not significantly adjacent to the SSc-associated genes. Phosphodiesterase-5 inhibitors, endothelin receptor blockers, and some of the immunosuppressive agents (sirolimus, tocilizumab and methotrexate), prostacyclin analogs (tranilast and iloprost), tyrosine kinase inhibitors (TyKIs: ninedanib, imatinib and dasatinib) and hydroxyfasudil were all found to be located significantly proximal to the SSc-associated genes (*z*_*c*_ <  −1.645, *P *value < 0.05).

### Proximity between SSc-targeted drugs and SSc-relevant pathways

To investigate further the network-based mechanism of drug action, we examined the pathways that are proximal to the SSc-targeted drugs using the gene sets of the KEGG [Kyoto Encyclopedia of Genes and Genomes] and Reactome pathways^20,21^. We curated the SSc-relevant pathways or processes from the literatures^22–26^. Figure 3b depicts the heatmap analysis for the SSc-relevant pathways (see the Supplementary Table 3 for the detailed values). TyKIs, such as dasatinib, imatinib and nintedanib, significantly covered the broadest range of the signaling and extracellular-matrix organizing pathways (*z*_*c*_ <  −1.645, *P *value < 0.05): toll-like receptor signaling pathway, chemokine-, JAK-STAT-, Fc-εRI-, B-cell receptor-, vascular endothelia growth factor (VEGF)-, mTOR-, platelet-derived growth factor (PDGF)-, type I and II interferon-signaling pathways and keratan sulfate/chondroitin sulfate/dermatan sulfate biosynthesis. Among the TyKIs, nilotinib showed the weakest accessibility to the SSc-relevant pathways. Nilotinib was defined to target only two tyrosine kinases, ABL1 and KIT, whereas the TyKIs listed above had 9 or 10 molecular targets (see the Supplementary Table 2). Endothelin receptor blockers were found to be located close to chemokine-, VEGF-, hypoxia-inducible factor (HIF)-1-, Apelin- and sphingolipid-signaling pathways (*z*_*c*_ < −1.645, *P *value < 0.05), but not significantly close to extracellular-matrix organizing processes, which is consistent with previous results^27–29^. Among the immunosuppressive agents, methotrexate, sirolimus and tocilizumab showed the potential to perturb extracellular-matrix organization by interfering with biosynthesis of glycosaminoglycans. Halofuginone, having two targets, COL1A1 and MMP2, was significantly close to extracellular-matrix organizing processes.

### Disease module identification

**Figure 4:**
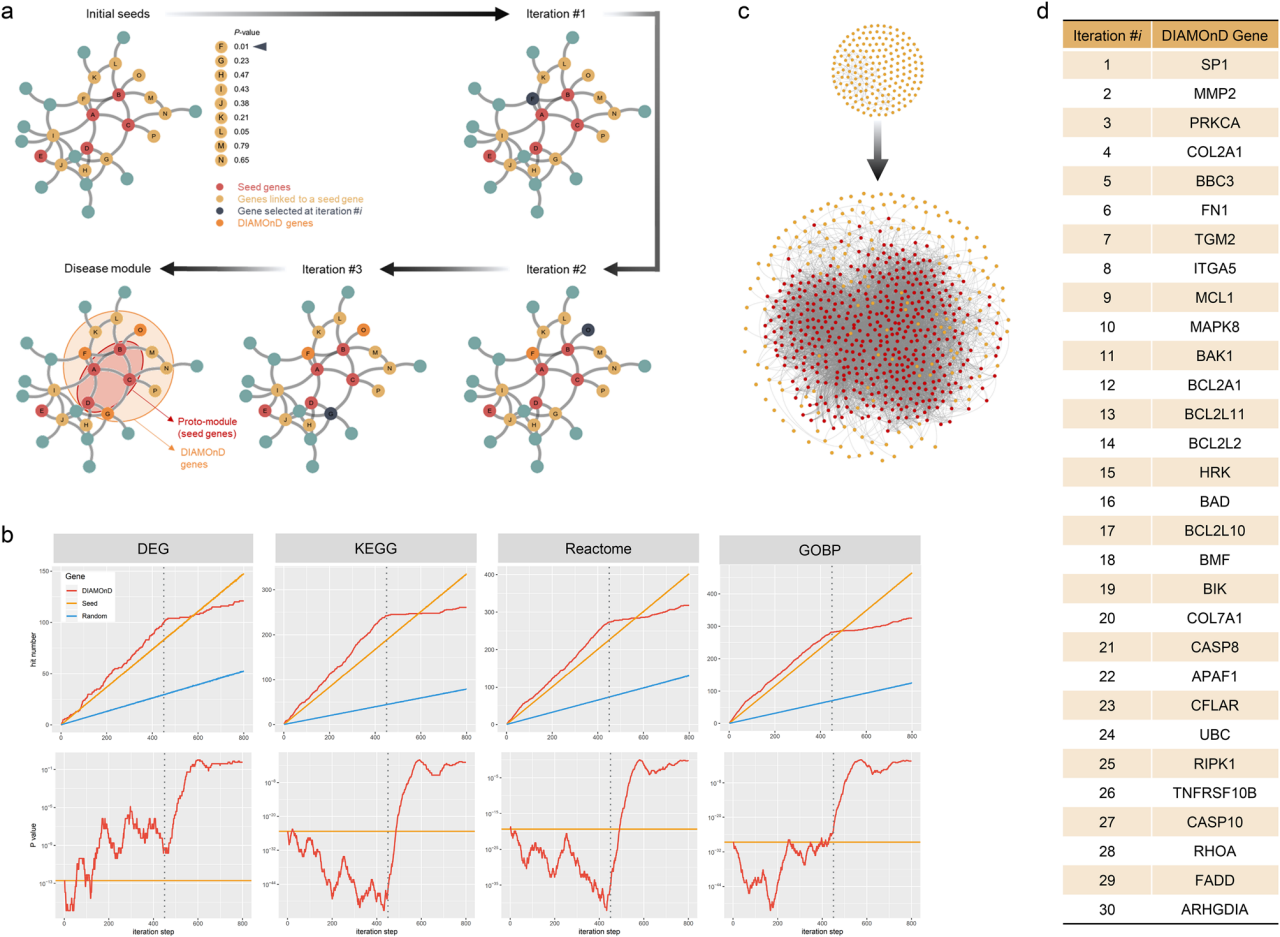
Disease-module identification and validation. (**a**) Schematic network configuration from seed genes to the SSc disease module. Seed genes (red) and their neighbor nodes (yellow) are identified iteratively. The proto-module consists of the seed genes only, whereas the final disease module incorporates all genes identified by the DIAMOnD method. (**b**) Validation of the identified genes through cross-reference with orthogonal data sources. The upper row depicts the number of DIAMOnD genes found in the different validation datasets (SSc gene expression data, gene sets from KEGG, Reactome pathways, and GO biological process terms), with the corresponding statistical significance (*P *value) shown on the lower row. No significant gain was noted beyond 450 iteration steps (vertical gray dotted lines). In the upper row, a sliding-window approach was used to adjust for the dependence of *P *values on the underlying set size. (**c**) The 179 seed genes and the resulting network once the 450 DIAMOnD-identified genes were added to create the 629-gene SSc disease module. (**d**) List of the DIAMOnD genes from the first 30 iterations.

Known disease-associated genes tend to be investigated most extensively, which might introduce a bias and, thereby, produce an incomplete explanation of the disease pathogenesis. To counter that limitation, we based the disease module on the established SSc-associated genes. A cluster of 88 highly interconnected seed genes was observed on the interactome of the 179 SSc-associated genes from three sources^12–14^ shown in Fig. 2a, which we called the “proto-module”. Proteins that are involved in the same disease tend to interact with each other^30^. We prioritized the putative SSc-relevant genes forming the disease module on the interactome using the Disease Module Detection (DIAMOnD) algorithm (Fig. 4a, Supplementary Table 4)^31,32^. The DIAMOnD algorithm prioritizes the proteins in the network based on their topological proximity to seed proteins. Hence, the order in which the DIAMOnD proteins are selected can be interpreted as a network-based ranking criterion. DIAMOnD ranks the entire network in consecutive order and we, therefore, needed an additional stopping criterion to define the boundary of the disease module. For this, we used four different SSc-specific validation details: human SSc gene expression data, gene-sets from the KEGG and Reactome pathways, and GO-biological process terms. The first item comprises the differentially expressed genes, and the three latter items were assembled from the significantly enriched pathways or processes, based on the SSc-associated seed genes. The DIAMOnD gene in each iteration was considered a “hit” if it was incorporated in the gene-sets. (Fig. 4b). There was no further significant gain beyond the 450th iteration in terms of hit rate and *P *value (Fig. 4b), so 450 DIAMOnD genes were added to the 179 seed genes to make the final SSc disease module (Fig. 4c). The top 30 hits are listed in Fig. 4d. The SSc disease module from the KEGG pathways was most strongly enriched with the MAPK-signaling pathway (*P *value = 2.85 × 10^−80^), PI3K-Akt-signaling pathway (*P *value = 8.09 × 10^−69^) and tumor necrosis factr (TNF)-signaling pathway (*P *value = 1.55 × 10^−64^), followed by the chemokine-signaling pathway (*P *value = 7.70 × 10^−42^), Fcε RI-signaling pathway (*P *value = 3.32 × 10^−45^) and the JAK-STAT signaling pathway (*P *value = 2.09 × 10^−40^).

### Perturbation of disease modules by drugs

**Figure 5:**
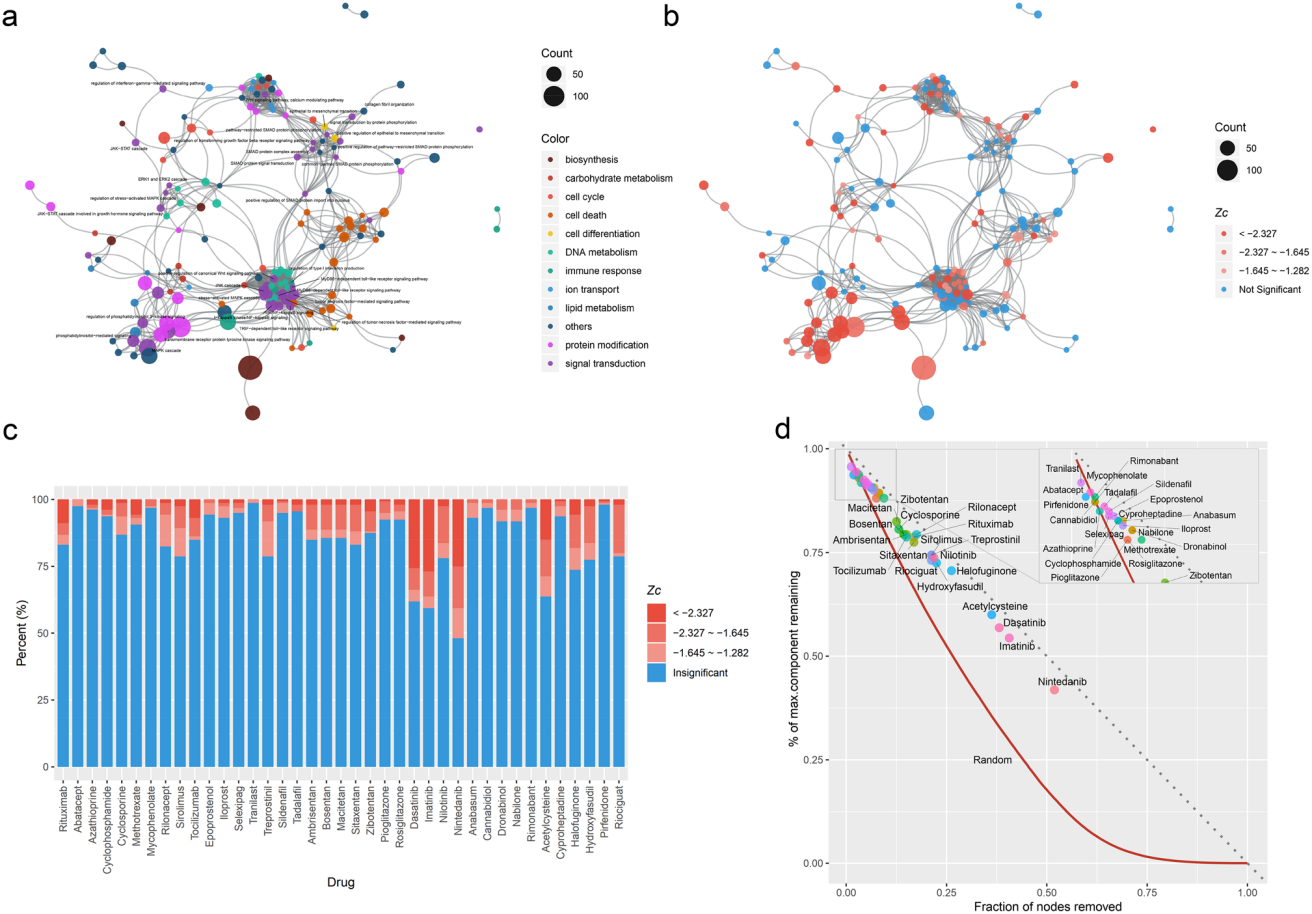
SSc disease module and perturbation by drugs. (**a**) Enriched pathway map of the SSc disease module. Nodes represent distinct GO terms. Node size is proportional to the number of genes in the respective GO term. The edge was determined by the Jaccard similarity coefficient (edge cut-off is 0.1 distance). (**b**) Perturbation of the disease module by nintedanib. Nodes significantly proximal to drug targets are red (*z* ≤ −1.282), insignificant nodes are blue (*z* > −1.282). If *z*_*c*_ ≤  −1.645 (one-sided *P *value < 0.05), a node was considered to be significantly proximal to the disease and if *z*_*c*_ ≤  −1.282 (one-sided *P *value < 0.10), a node was considered to be probably proximal to the disease. (**c**) The ratio of nodes, based on the proximity between the drugs and the disease module, indicates the specificity and generalization of the respective drugs. (**d**) The percentage of the largest connected component (LCC) that remains when treated by drugs (points) or under random node failure (red line, 10^3^ simulations).

To understand the pathways or cellular processes, as guided by the disease module, we performed functional enrichment analysis for all seed and DIAMOnD genes, using the DAVID software^15^ and constructed the enrichment map33. The graph of enriched GO-biological processes was made up of 160 nodes with 1,086 edges, and the LCC comprised 155 nodes (Fig. 5a and Supplementary Fig. 1). To check how close a drug was to each node enough to perturb the process, we calculated the network-based proximity of drug targets to the gene set of each node in the same way for disease and pathway proteins. We regarded the node proximal to a certain drug (*z*_*c*_ < −1.282, *P *value < 0.10) as being perturbed.

In an attempt to understand the mechanisms of action of the respective drugs, we analyzed the biological processes and molecular functions of the GO terms in the disease network. To illustrate, nintedanib, the most perturbing agent for this network, is cited as an example. The terms most proximal to nintedanib were ‘JAK-STAT cascade’ (*z*_*c*_ = −6.11, *P *value < 1 × 10^−5^), ‘regulation of interferon-γ-mediated signaling pathway’ (*z*_*c*_ = −5.45, *P *value < 1 × 10^−5^), ‘regulation of phosphatidylinositol 3-kinase signaling’ (*z*_*c*_ = −4.87, *P *value < 1 × 10^−5^), and ‘TNF-mediated signaling pathway’ (*z*_*c*_ = −4.42, *P *value < 1 × 10^−5^). Nintedanib perturbed 83 (51.8%) nodes, which are shown in red in Fig. 5b. The distribution of nodes perturbed by each drug is provided in Fig. 5c and Supplementary Fig. 2.

In a network context, robustness refers to the system’s ability to carry out its basic functions even with the breakdown of some network nodes or links. The connectivity of the LCC is regarded as the foundation of robustness^34^. To investigate the robustness of the disease-module network, we computed the evolution of the size of the LCC under random failures of network nodes (Fig. 5d)^34,35^. Our results (1,000 simulations) argue that half of the network would collapse when at least 27% of the nodes are disturbed, on average. Nintedanib showed the most perturbing activity (51.8%), followed by imatinib (40.6%), dasatinib (38.1%) and acetylcysteine (36.2%).

### Clinical response in SSc patients receiving nilotinib therapy

**Figure 6:**
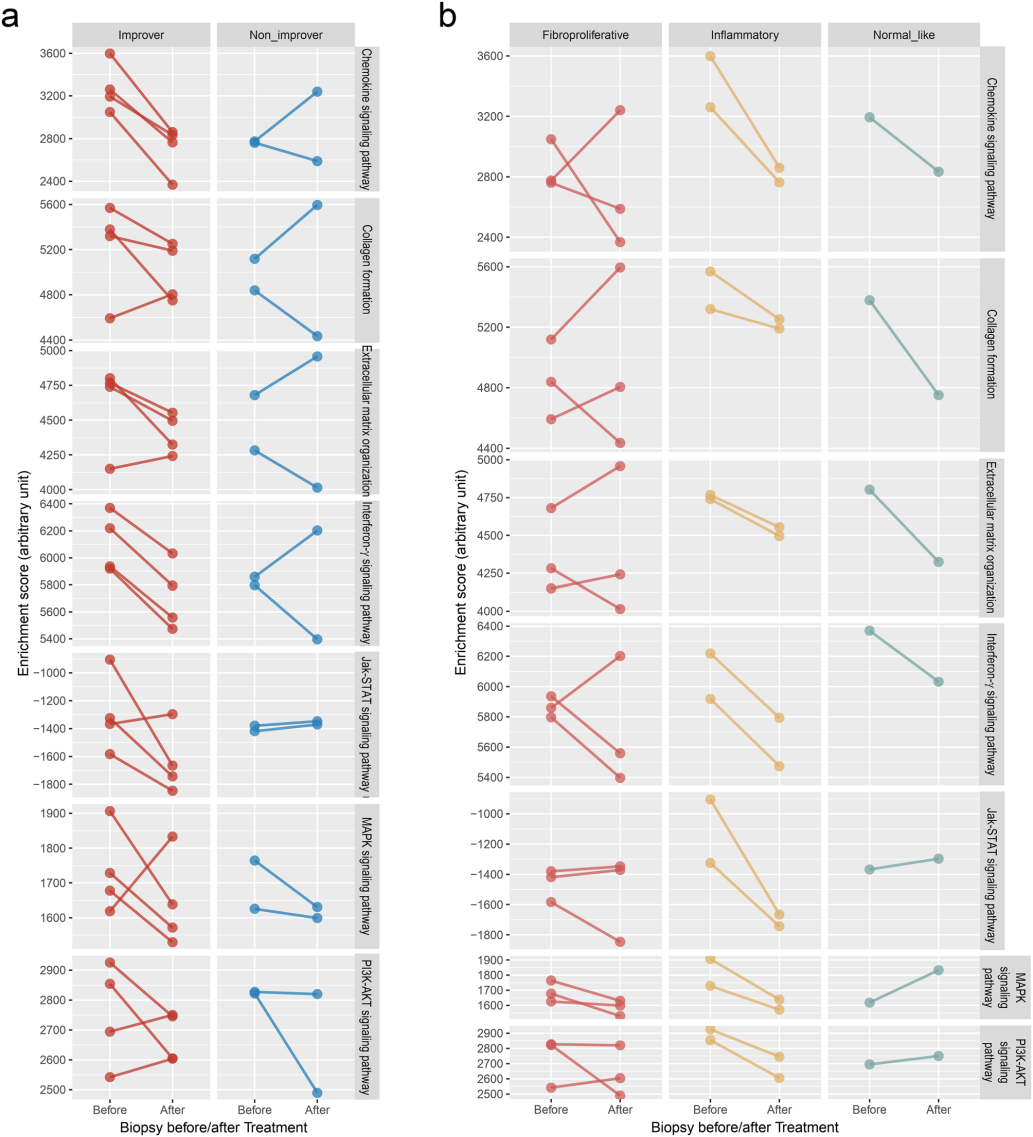
Validation of network-based proximity of nilotinib. ssGSEA was performed for paired skin biopsy samples obtained from SSc patients receiving 12 months of nilotinib treatment (GSE65405). The enrichment scores of seven main signaling pathways or processes were compared for before- and after-treatment. (**a**) Comparison by improvers versus non-improvers. Improvers were defined as those patients with a decrease in modified Rodnan skin score of > 20% from baseline to 12 months. (**b**) Comparison by intrinsic molecular subsets. Statistical tests were not possible due to the small number of samples.

To test the applicability of network-based proximity of drugs, we imported the gene expression dataset of SSc skin tissues, which was obtained from 6 SSc patients on 2 separate occasions, before and after 12 months of treatment with nilotinib (GSE65405)^36^. To compare the paired samples before and after nilotinib treatment, a single sample version of gene-set enrichment analysis (ssGSEA) was used to generate enrichment scores for gene sets of SSc-relevant pathways or processes. Patients with a decrease in modified Rodnan skin score > 20% from baseline to 12 months were defined as improvers^36^. An intrinsic molecular subset was assigned according to the previous study^37^, and three fibroproliferative, two inflammatory, and one normal-like subsets were identified. All patients in the inflammatory and normal-like subsets, and in one of the three fibroproliferative subsets were classified as improvers. Seven main signaling pathways or processes targeted by nilotinib on the SSc disease-module network were compared for the improvement and intrinsic subsets (Fig. 6). A decreasing pattern was found in most of the improvers (Fig. 6a), and was particularly notable in the two inflammatory subsets (Fig. 6b).

## Discussion

Advances in high-throughput platforms, the continuing accumulation of knowledge about diseases, and the availability of organized biomedical data add to the clinical and mechanistic insights into disease from a wide and systemic perspective^38,39^. The resolution of tissue fibrosis remains an unmet clinical need in patients with SSc. In the present study, we tried to understand the disease and currently used and potential drugs’ action in terms of network biology, to reduce the gaps in the existing knowledge, and to provide clues into the challenging issue in SSc.

One hundred seventy-nine SSc-associated genes were identified from the database. Eighty-eight molecules constituted the largest subnetwork and others were sparsely connected or isolated on the interactome. This contributed to an incomplete interactome, false positives and missing disease genes^32^. Moreover, enriched processes of those genes were limited to chemokine synthesis, apoptosis-associated processes, the TGF-β signaling pathway, extracellular-matrix organization, and some immune response-related processes (MHC class II biosynthesis process and T-cell proliferation), and they do not fully cover the current knowledge about SSc. To make up for the lack of completeness, we reconstituted the SSc disease module using the DIAMOnD algorithm, and the final SSc disease module and enriched processes better explained the pathophysiology of SSc. It is noteworthy that SP1^40^, FN1^41^, and apoptosis-related molecules including BCL2 family^42^, the emerging small molecular targets of SSc, were incorporated early by priority into DIAMOnD genes, although those were not assigned as seed genes. In particular, it is notable that inhibition of BCL-X_L_ reversed the established fibrosis in a mouse model of SSc by inducing myofibroblast apoptosis, and that that provided a new therapeutic avenue for treating SSc^42^. Taken together, this principle of disease gene prioritization might be applicable to discovering novel or critical molecules involved in the development of SSc but not well-defined before.

To understand the action of drugs and their status with relation to SSc on the interactome, the proximity between drug targets and disease genes was evaluated. Proximity between the currently used or potential drugs and disease genes was variable and not all of the proximity value showed statistical significance. Non-significant closeness would mean that the drug’s targets are located far from the disease module or that the drug has an indirect effect on the disease module. It could be also ascribed to its different target among the etiopathogenetic domains (immune-inflammatory, vascular, or fibrotic process) or to its limited indication for a specific clinical manifestation (interstitial lung disease, pulmonary arterial hypertension or cutaneous involvement). Proximity of the pathways offered a complementary explanation for this difference. Endothelin receptor blockers exhibited a good proximity to disease genes as a whole, but were limited to several key pathways such as VEGF- and HIF-1-signaling pathways. In contrast, TyKIs, especially those with multiple targets, approached disease genes through multiple pathways, including both inflammatory and extracellular-matrix organizing processes. Even as a control, statins showed a fair proximity to disease genes and several pro-fibrosing pathways. Statins have been reported to be associated with the development and exacerbation of lung fibrosis^43,44^.

We investigated the size and range of the disease module differentially covered by each drug, to understand its mode of action and topology in the network-based framework. We noticed variable sizes and differential coverage of the disease network by the drugs. This helped provide a comprehensive view of each drug’s action and repercussions within the disease module. As a single agent, nintedanib showed the best performance in perturbing the disease module, followed by imatinib, dasatinib, and acetylcysteine. Acetylcysteine, a precursor of the antioxidant glutathione, was defined to target ACY1, CHUK, GSS, IKBKB, SLC7A11 and the glutamate receptor family. In a randomized controlled trial, the addition of acetylcysteine to prednisone and azathioprine worked in preserving pulmonary function in patients with idiopathic pulmonary fibrosis^45^ although this is not generally recommended due to the concern for harmful effects^46^. Acetylcysteine may be more beneficial in individuals with idiopathic pulmonary fibrosis with specific genotype such as TOLLIP^47^. This approach can also be applied to estimation of the combined or synergistic effects across drugs. Combinations of two drugs could create a synergistic or additive effect if their coverage for the disease-module network is complementary to each other, and could increase the success rates of drug repositioning^48^.

TyKIs were promising agents because experimental studies in murine models of SSc have shown that TyKIs can prevent the development of fibrosis^16^, and this was corroborated by our network-based simulation. But discrepancies exist between encouraging preclinical results and disappointing clinical trials^11^. A vital clue can be obtained from a pilot study using the gene expression dataset of SSc skin tissues, which was obtained from 6 SSc patients receiving 12 months of nilotinib treatment^36^. Effective suppression of the SSc-relevant pathways and good response alleviating the skin fibrosis was observed in the inflammatory subsets of SSc patients. This implied that TyKIs are more effective in on-going pro-fibrotic inflammation and less in the fibroproliferative phase over the threshold. Lower effectiveness could partly be attributed to the lower dose (400 mg qd, rather than 400 mg q12 hour for chronic myeloid leukemia)^36^, the narrow selectivity of nilotinib targets and the low levels of the target molecules^49^. Unfortunately, toxicity and tolerability issues limited the further use of imatinib and nilotinib^11^. Taken together, TyKIs with less selectivity and a broad range of tyrosine kinase targets, such as dasatinib and nintedanib, would have greater efficacy and less toxicity, especially in the subset of SSc patients in the early inflammatory phase. This could guide clinical trial design and subgroup analysis.

Our results argue that network-based drug-disease proximity offers a novel perspective into a drug’s therapeutic effect in the refined disease module. In our network-based model, many drugs failed to have a comprehensive capability to subdue the SSc disease module although they are able to perturb a few key pro-fibrosing pathways. This limitation could be overcome with combination therapy or the use of drugs with multifarious activities, like TyKIs. In this work, we investigated just one of the dimensions that need to be taken into account when determining the feasibility of drugs for each case, as the actual clinical outcome is determined by a plethora of variables, including disease status, genetic susceptibility, comorbidity, pharmacokinetics and/or pharmacodynamics. Significant additional research is needed to translate these findings into clinical applications.

## Methods

### The human interactome

A previous study^30^ constructed a model of the human interactome based on its protein–protein interaction (PPI) network built based on the experimentally documented human physical interactions from the reputable database^21,50–57^, as well as a separately published large-scale signaling network^58^. This human interactome dataset contains 141,150 interactions among 13,329 proteins, and was found to perform better in capturing the therapeutic effect of drugs than did functional networks from STRING^59^ and other high-throughput binary screens^8^. We used this human interactome network as the basis for conducting our proximity analysis.

### Systemic sclerosis-associated genes and drug targets

SSc-associated genes were retrieved from PheGenI^12^, DisGeNET^13^, and CTD^14^. Forty-five, 70 and 105 genes were found to be associated with SSc in PheGenI, DisGeNET, and the CTD, respectively. One hundred seventy-nine unique genes belong to human interactome elements (Supplementary Table 1). Information on drug-target molecules was extracted from the DrugBank database (Supplementary Table 2)^19^.

### Network-based proximity measure

To quantify the network-based relationship between drug targets and disease proteins, we used the metric of *closest proximity d*_c,_ which is defined as the average shortest path-length between the drug’s targets and the nearest disease protein^8^. Briefly, given *S*, the set of disease proteins, *T*, the set of drug targets and *d(s, t)*, the shortest path-length between nodes *s* and *t* in the network, *d*_c_ between S and T is given by:

$$ d_{{c\left( {S,T} \right)}} = \frac{1}{T}\mathop \sum \limits_{t \in T} m{\text{i}}n_{s \in s} {\text{d}}\left( {s,t} \right) $$

To evaluate the significance of the network distance between a drug and a given disease, we constructed a reference distance distribution corresponding to the expected distance between two randomly selected groups of proteins of the same size and degree distribution as the original disease proteins and drug targets in the network. This procedure was repeated 1,000 times. The mean *d* and standard deviation (*σ*_*d*_) of the reference distribution were used to calculate a z-score (*z*_*d*_) by converting an observed (non-Euclidean) distance to a normalized (non-Euclidean) distance. The detailed description of network proximity was provided in the previous study^8^. According to the standard normal distribution, if *z*_c_ ≤  −1.645 (one-sided *P *value < 0.05), a drug was considered to be *significantly proximal* to the disease and if *z*_c_ ≤  −1.282 (one-sided *P* value < 0.10), a drug was considered to be *probably proximal* to the disease.

### Network-based pathway proximity analysis

To identify the biological pathways affected by a drug in the human interactome, we used the closest distance measure to assess the proximity between drugs and pathways. The drug pathway proximity is the normalized distance between the drug targets and the proteins belonging to a given pathway. As in our calculation of drug-disease proximity, 1,000 randomly selected protein sets, matching the original protein sets in size and network degree, were used to calculate the mean and the standard deviation of the *z*-score. We used gene sets from all of the KEGG^21^ and Reactome pathways^20^.

### Disease module detection method

To derive the SSc-specific disease module in the human interactome, we used the Disease Module Detection (DIAMOnD) algorithm^31,32^, which is robust over a wide range of noise and shows superior performance relative to other methods. This algorithm is based on the observation that potential disease genes have a propensity to interact with the known disease genes, and prioritizes the proteins in the network based on their topological proximity to seed proteins^30^. The detailed description of DIAMOnD algorithm was provided in the previous studies^31,32^.

This analysis identified the statistically significant enrichment of SSc-relevant genes among the designated DIAMOnD genes. Because there was no significant further gain beyond 450 iterations, we considered the first 450 DIAMOnD genes to have the most significant SSc association (Supplementary Table 4). The module, the LCC in the network, which started at 88 directly connected seed genes, was incremented by 450 DIAMOnD genes and was further connected to 62 isolated seed genes through the link by DIAMOnD genes. As a result, a putative SSc disease module of 600 genes, in total, was created.

### Validation of DIAMOnD genes

To determine the cut-off size of DIAMOnD genes, we used four SSc-relevant validation datasets: the SSc gene expression data, gene sets from KEGG and Reactome pathways and GO biological process terms. To obtain RA gene expression data, we searched the NCBI Gene Expression Omnibus (GEO, January 2018, https://www.ncbi.nlm.nih.gov/geo/) and ArrayExpress database (https://www.ebi.ac.uk/arrayexpress/) for all gene expression datasets using ‘systemic sclerosis’ and ‘skin tissue’ as a search term, resulting in eight publicly available human microarray datasets. Because there was a trade-off between the number of studies to include and the number of genes that are within the intersection from all datasets, we optimized the product of the two by selecting the point where those two trends crossed. We finally selected 8 expression datasets with GEO series (GSE) IDs: GSE58095, GSE76806, GSE66321, GSE65405, GSE32413, GSE45485, GSE59785 and GSE76807. The complete collection included 423 skin samples from 175 SSc patients and 61 healthy controls. After normalizing and removing the batch effect, we performed differential expression analysis and selected the gene candidates that were identified in each of three independent methods (empirical Bayes, significance analysis of microarrays, and ranked product), and finally filtered the 2,175 up-regulated genes. The gene sets of KEGG, Reactome pathways and GO terms that were significantly enriched within the given set of seed genes were obtained using the Database for Annotation, Visualization, and Integrated Discovery (DAVID) version 6.8 software and the Reactome Analysis tool (*P *value < 0.05)^15,20^. Each DIAMOnD gene was considered a “hit” if it had at least one annotation that was in the gene sets significantly enriched within the seed genes (Fisher’s exact test, *P *value < 0.05). To compensate for the dependence of *P *values on the underlying set size, we used a sliding-window approach: at each iteration step i, we considered all DIAMOnD genes in the interval [i − 179/2, i + 179/2], thereby obtaining sets of the same size as the seed genes, which could be compared with each other.

### Assessment of network robustness

To assess the robustness of the networks against attack, we used iterative ‘random’ and ‘targeted’ removal of nodes^34,35^. Nodes were deleted randomly from the graph irrespective of the degree of the node, or only the nodes targeted by the drugs were selectively removed. After each deletion, we computed the evolution of the size of the LCC, which means the minimum path-length under a random or targeted attack of a graph. This indicates how much the drug can disrupt the network of the disease module.

### Functional enrichment analysis

We performed functional enrichment analysis focusing on the genes of interest using DAVID software^15^. Terms were considered to be significant if the *P* value was less than 0.01, the gene count more than 3 and the fold enrichment larger than 1.5. The Expression Analysis Systematic Explorer (EASE) score was transformed using the absolute base-10 logarithm of the *P* value. The enrichment results were visualized with the Enrichment Map format, where nodes represent gene sets and weighted links between the nodes represent an overlap score, which depends on the number of genes that the two gene sets share (Jaccard coefficient)^33^. To intuitively identify redundancies between gene sets, the nodes were connected if their contents overlapped by more than 10%.

### Single sample gene-set enrichment analysis

To test for gene enrichment in individual samples from SSc patients, we used a single sample version of gene-set enrichment analysis (ssGSEA), which defines an enrichment score as the degree of absolute enrichment of a gene set in each sample within a given dataset^60^. The gene expression values for a given sample were rank-normalized and an enrichment score was produced using the Empirical Cumulative Distribution Functions of the genes in the signature and the remaining genes. This procedure is similar to the GSEA technique, but the list is ranked by absolute expression in one sample.

### Statistical analysis

For continuous distributed data, between-group comparisons were performed using a paired or unpaired *t*-test. Categorical or dichotomous variables were compared using a chi-squared test or Fisher’s exact test. All analyses were conducted in *R* (version 3.5.2, The R Project for Statistical Computing, www.r-project.org).

## Supplementary information

Supplementary information

Supplementary Table 1.

Supplementary Table 2.

Supplementary Table 3.

Supplementary Table 4.

## Data Availability

The SSc skin transcriptomic datasets used in this study are freely available on the GEO data portal under the access GSE58095, GSE76806, GSE66321, GSE65405, GSE32413, GSE45485, GSE59785 and GSE76807.
