# Author Correction: Network-based modeling of drug effects on disease module in systemic sclerosis

**DOI:** 10.1038/s41598-021-87277-w

**Published:** 2021-04-09

**Authors:** Ki-Jo Kim, Su-Jin Moon, Kyung-Su Park, Ilias Tagkopoulos

**Affiliations:** 1 Division of Rheumatology, Department of Internal Medicine, St. Vincent’s Hospital, College of Medicine, The Catholic University of Korea, Seoul, Republic of Korea; 2 Division of Rheumatology, Department of Internal Medicine, Uijeongbu St. Mary’s Hospital, College of Medicine, The Catholic University of Korea, Seoul, Republic of Korea; 3 St. Vincent’s Hospital, 93 Jungbu‑daero, Paldal‑gu, Suwon, Gyeonggi‑do 16247 Republic of Korea; 4 Department of Computer Science, University of California, Davis, CA USA; 5 Genome Center, University of California, Davis, CA USA; 6 AI Institute for Next-Generation Food Systems, AIFS, Davis, CA USA

Correction to: *Scientific Reports* 10.1038/s41598-020-70280-y, published online 07 August 2020

Ilias Tagkopoulos was omitted from the author list in the original version of this Article. Furthermore, Ilias Tagkopoulos was omitted as a corresponding author. Correspondence and request for materials should also be addressed to iliast@ucdavis.edu

The Author Contributions section now reads:

“K-J Kim and I Tagkopoulos conceived the idea and designed the study. Ki-Jo Kim and S-J Moon carried out data collection. K-J Kim performed the computational analysis. K-J Kim, S-J Moon, and K-S Park analyzed and interpreted the data. K-J Kim, K-S Park, and I Tagkopoulos wrote the manuscript, and all authors contributed to its revision. I Tagkopoulos supervised all aspects of the project. All authors read and approved the final manuscript.”

This has been corrected in the PDF and HTML versions of the Article, and in the accompanying Supplementary Information file.

