# Supplementary material for: Network-based modeling of drug effects on disease module in systemic sclerosis
Source: Sci Rep. 2020 Aug 7;10:13393. doi: 10.1038/s41598-020-70280-y (PMC7414841; doi:10.1038/s41598-020-70280-y)
Supplement: Supplementary file 1 — Supplementary information [file 41598_2020_70280_MOESM1_ESM.pdf]

Network-based modeling of drug effects on disease module in systemic sclerosis

Ki-Jo Kim[1,3], Su-Jin Moon[2], Kyung-Su Park[1], Ilias Tagkopoulos[4,5,6]

[1] Division of Rheumatology, Department of Internal Medicine, St. Vincent's Hospital, College of Medicine, The Catholic University of Korea, Seoul, Republic of Korea

[2] Division of Rheumatology, Department of Internal Medicine, Uijeongbu St. Mary's Hospital, College of Medicine, The Catholic University of Korea, Seoul, Republic of Korea

[3] St. Vincent's Hospital, 93 Jungbu-daero, Paldal-gu, Suwon, Gyeonggi-do 16247, Republic of Korea.

[4] Department of Computer Science, University of California, Davis

[5] Genome Center, University of California, Davis

[6] AI Institute for Next-Generation Food Systems, AIFS, Davis, CA

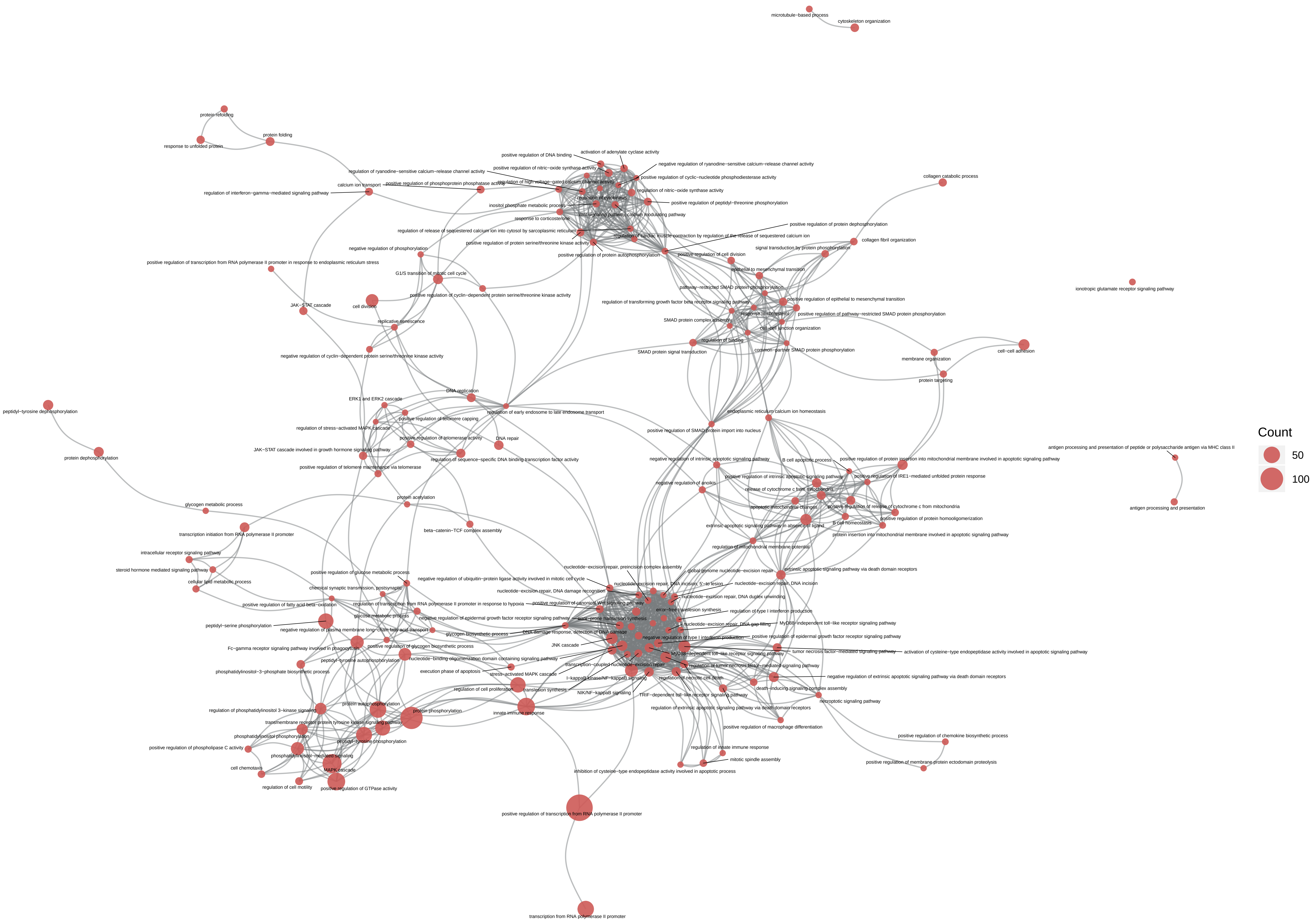

Figure S1. Gene-Set enrichment map for SSc disease module reconstituted using DIAMOnD algorithm. Same as **Figure 5A** except for detailed annotation.

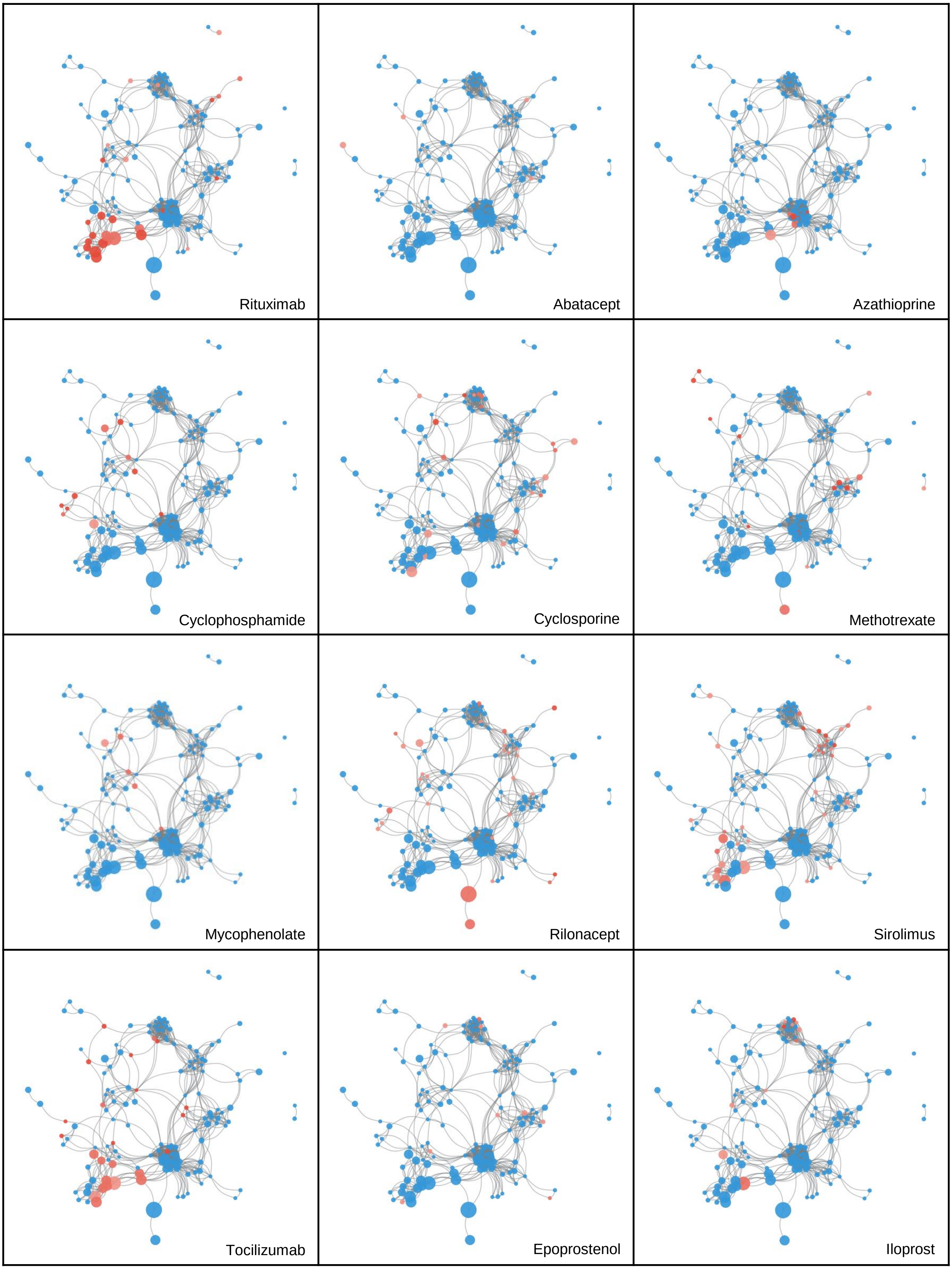
Rituximab
Abatacept
Azathioprine
Cyclophosphamide
Cyclosporine
Methotrexate
Mycophenolate
Rilonacept
Sirolimus
Tocilizumab
Epoprostenol
Iloprost

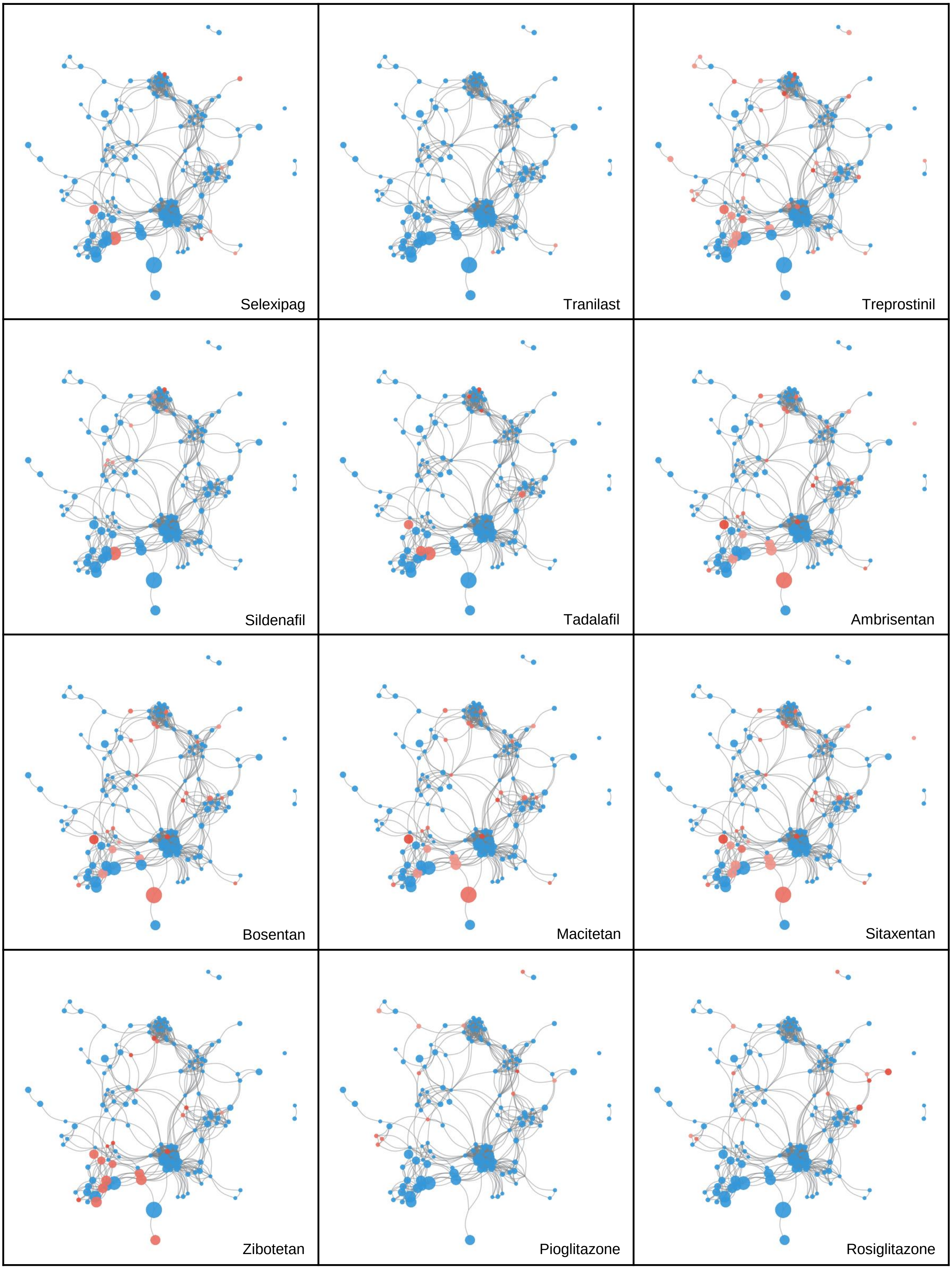

Selexipag
Tranilast
Treprostinil
Sildenafil
Tadalafil
Ambrisentan
Bosentan
Macitetan
Sitaxentan
Zibotetan
Pioglitazone
Rosiglitazone

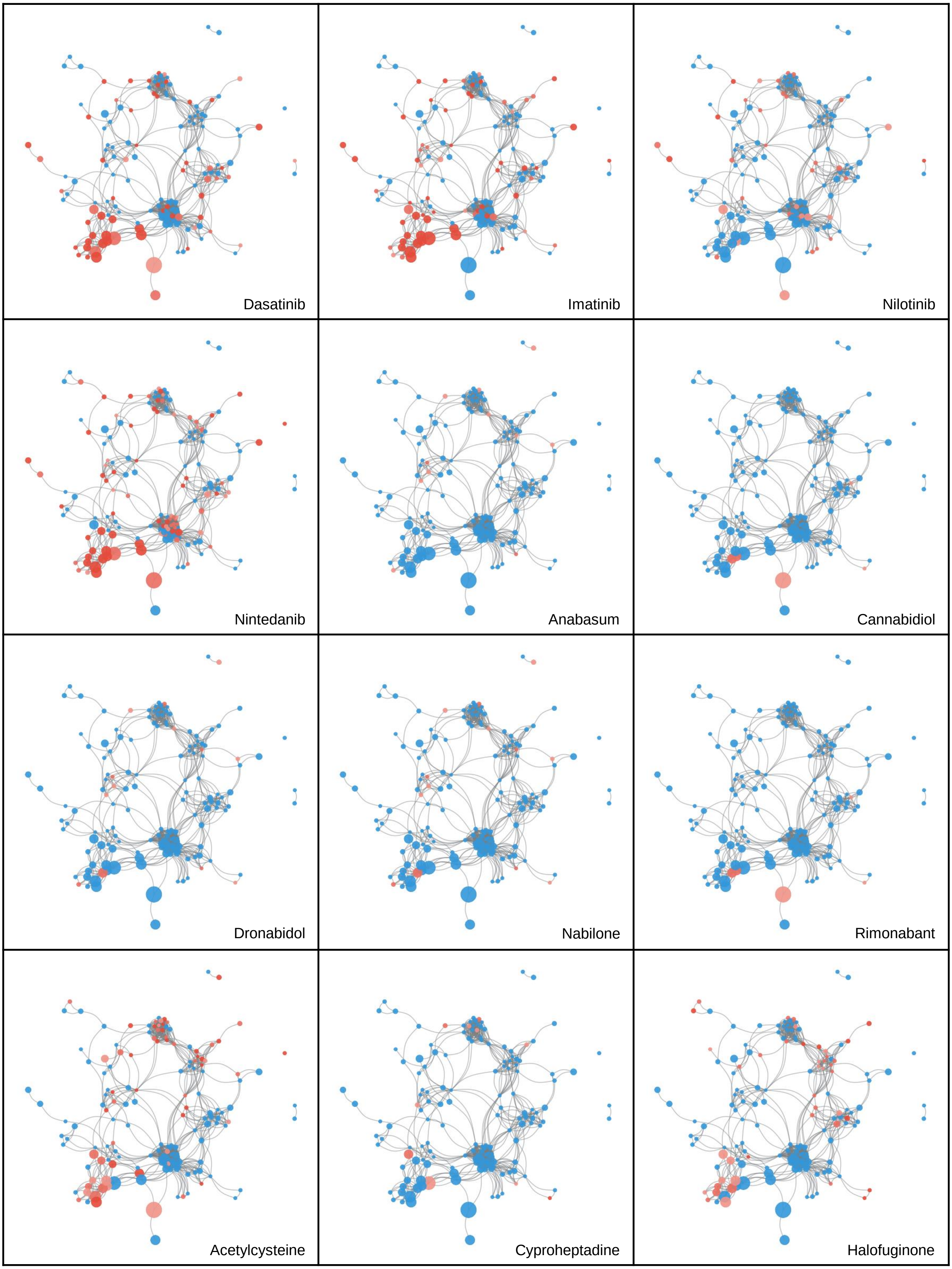
Dasatinib
Imatinib
Nilotinib
Nintedanib
Anabasum
Cannabidiol
Dronabidol
Nabilone
Rimonabant
Acetylcysteine
Cyproheptadine
Halofuginone

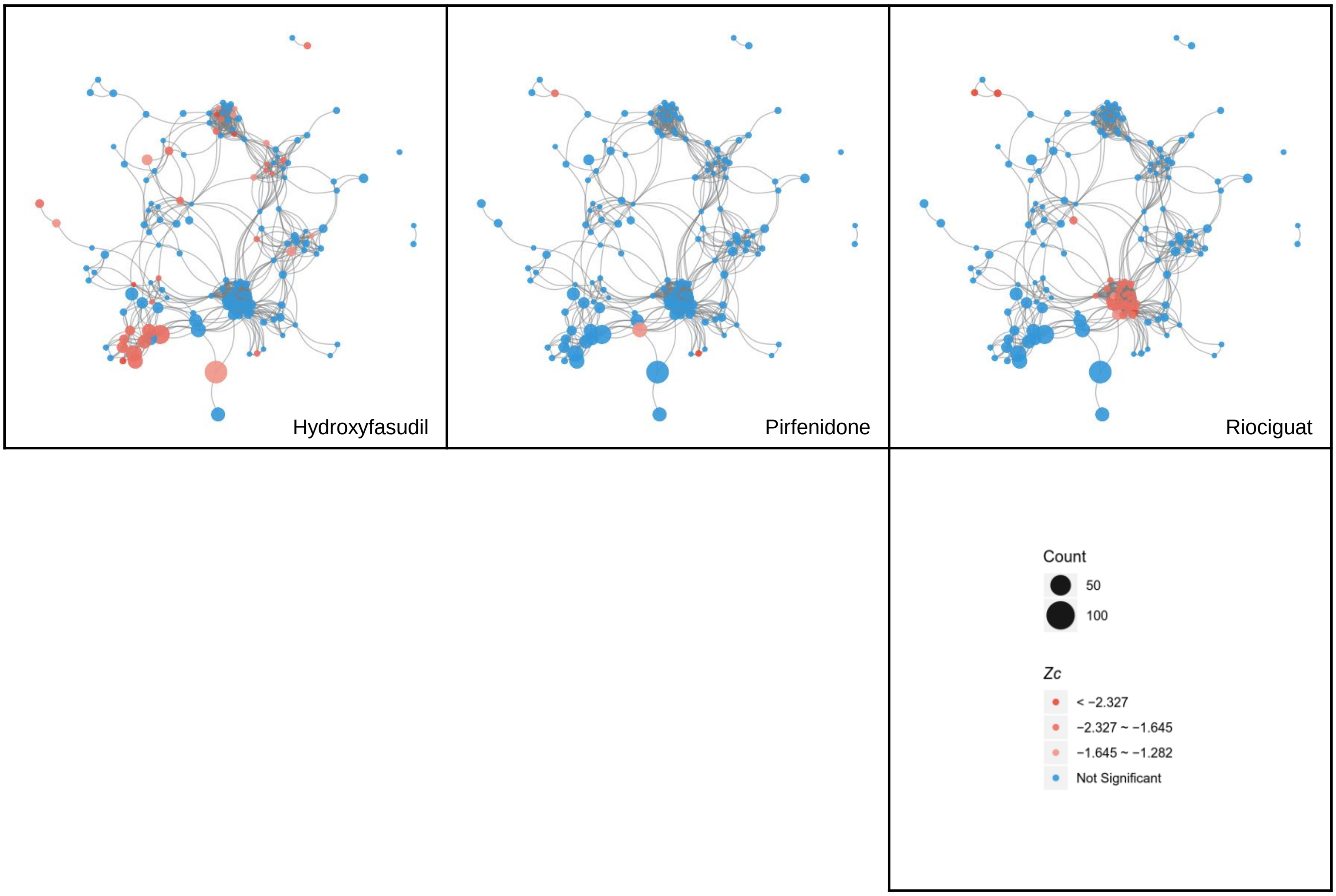

Figure S2. Network perturbation by proximity between drugs and disease module. Layout of the network is the same as the enrichment map of SSc disease module (**Figure 5A**). Nodes significantly proximal to drug targets are colored by red hue depending on the degree of proximity and insignificant nodes are coated by blue color.
